# Characterization and therapeutic perspectives of differentiation-inducing therapy in malignant tumors

**DOI:** 10.3389/fgene.2023.1271381

**Published:** 2023-09-08

**Authors:** Kangwei Zhu, Yuren Xia, Xindi Tian, Yuchao He, Jun Zhou, Ruyu Han, Hua Guo, Tianqiang Song, Lu Chen, Xiangdong Tian

**Affiliations:** ^1^ Key Laboratory of Cancer Prevention and Therapy, National Clinical Research Center for Cancer, Tianjin’s Clinical Research Center for Cancer, Tianjin Medical University Cancer Institute and Hospital, Tianjin, China; ^2^ Department of Biofunction Research, Institute of Biomaterials and Bioengineering, Tokyo Medical and Dental University (TMDU), Chiyoda, Japan

**Keywords:** cell differentiation, differentiation-inducing therapy, cell cycle, retinoic acid, differentiation mechanisms

## Abstract

Cancer is a major public health issue globally and is one of the leading causes of death. Although available treatments improve the survival rate of some cases, many advanced tumors are insensitive to these treatments. Cancer cell differentiation reverts the malignant phenotype to its original state and may even induce differentiation into cell types found in other tissues. Leveraging differentiation-inducing therapy in high-grade tumor masses offers a less aggressive strategy to curb tumor progression and heightens chemotherapy sensitivity. Differentiation-inducing therapy has been demonstrated to be effective in a variety of tumor cells. For example, differentiation therapy has become the first choice for acute promyelocytic leukemia, with the cure rate of more than 90%. Although an appealing concept, the mechanism and clinical drugs used in differentiation therapy are still in their nascent stage, warranting further investigation. In this review, we examine the current differentiation-inducing therapeutic approach and discuss the clinical applications as well as the underlying biological basis of differentiation-inducing agents.

## 1 Introduction

Cancer is a major public health issue globally, and is one of the leading causes of death in both developing and developed countries (Bray et al., 2021; Sung et al., 2021). According to the Global Cancer Statistics 2020, 19.3 million new cases of cancer were projected worldwide, of which 10.0 million died (Sung et al., 2021). In developed countries, taking the United States as an example, 1,958,310 new cancer cases and 609,820 cancer deaths are estimated to occur in 2023 (Siegel et al., 2023). In a developing country like China, for instance, the results indicated that 4,292,000 new cancer cases and 2,814,000 cancer deaths were estimated to occur in 2015 (Chen et al., 2016). The cancer burden has risen globally, of which is expected to be 28.4 million cases in 2040 (Sung et al., 2021). Although available treatments, including surgery, chemotherapy, radiation, targeted therapy and immunotherapy, improve the survival rate of some cases, many advanced tumors are insensitive to these treatments (Qiu et al., 2021). Besides, traditional therapies would destroy the normal cells and tissues inevitably, which produce serious toxic side effects (Jin et al., 2020). Cancer cell differentiation involves reverting the malignant phenotype back to its original state, with the potential for trans differentiation to cell types characteristic of other tissues. Utilizing differentiation or trans differentiation as a strategy in high-grade tumor masses can offer a less aggressive approach to restrict tumor progression and enhance sensitivity to chemotherapy. A prime illustration of success is seen in the treatment of acute promyelocytic leukemia (APL), where the combination of retinoic acid (RA) and arsenic has achieved remarkable curative outcomes. Compared to traditional treatment, differentiation inducing therapy induces malignant cells re-differentiate into normal cells, without killing cells, avoiding toxic and side effects (Jin et al., 2020).

Cancer cell growth is characterized by a dysfunction in the normal process of cell proliferation, apoptosis, and terminal differentiation. The activation of specific pathways in normal cells induces cell differentiation, the process of acquiring phenotypic characteristics of mature fate, accompanied by a cessation of proliferation (Coffman, 2004; Kawamata et al., 2003; Kawamata et al., 2004). This abnormal differentiation results in rapid malignant proliferation of cancer cells. Differentiation-inducing therapy has been demonstrated to be effective in a variety of tumor types. Although an appealing concept, the mechanism and clinical drugs used in differentiation-inducing therapy are still in their nascent stage, warranting further investigation.

In this review, we examine the current differentiation-inducing therapeutic approach and discuss the clinical applications as well as the underlying biological basis of differentiation-inducing agents.

## 2 Overview of differentiation-inducing therapy

### 2.1 Relationship between differentiation and tumors

Differentiation ensures the specificity of morphology, function, and other characteristics in a developing cell. Dedifferentiation implies a dysfunction in cells and is characterized by loss of unique cell structure and function, owing to which cells transform into undifferentiated cells; for instance, normal stem cells differentiate abnormally to form tumor cells. Reportedly, differentiation arrest of the neural-crest-derived sympathoadrenal lineage has been found to result in neuroblastoma (NB) formation. Similarly, abnormal differentiation in hepatocyte progenitor cells result in hepatocellular carcinoma (HCC) and intrahepatic cholangiocarcinoma (ICC) with markers of progenitor cells (Sia et al., 2017; Zeineldin et al., 2022). Depending on the degree of tissue differentiation, tumors can be classified as highly, intermediately, and poorly differentiated carcinoma.

It is crucial to assess the status of solid tumor differentiation because undifferentiated histology is typically associated with tumor aggressiveness and poor prognosis. In this aspect, cell lines of various human solid tumors, such as NB, glioma, retinoblastoma (RB), pheochromocytoma, breast cancer, colon cancer, lung cancer, pancreatic cancer, cervical cancer, and laryngeal cancer, as well as the induction of cancer cell differentiation through differentiation inducers provide some information on the targets and markers for future differentiation-inducing therapy (Ebert and Salcman, 1994; Fassina et al., 1997; Guzhova et al., 2001; Munster et al., 2001; Vaudry et al., 2002; Lévy et al., 2003).

### 2.2 Origin of differentiation-inducing therapy

There are some connections in differentiation between normal cells and malignant tumor cells. For instance, hepatocytes are proposed to dedifferentiate into hepatocyte precursor cells, which then transform into HCC cells. At the same time, the hepatocyte precursor cells transdifferentiate to form the ICCs (Sia et al., 2017). This may explain the context-dependent consequences of differentiation on cellular processes and a better understanding of such mechanisms can help selectively target dedifferentiation for therapeutic purposes.

In the context of differentiation therapy, hormones or cytokines have been found to promote differentiation *in vitro*, thereby irreversibly modifying the phenotype of cancer cells (de Thé, 2018). In oncology, differentiation-inducing therapy refers to reactivating the differentiation of tumor cells via pharmacological intervention, which eventually results in tumor cell maturation and loss of malignant characteristics (Lotem and Sachs, 1988). Pathologists first noticed that tumor cells resembled immature cells in developing tissues a century ago, when they discovered a link between tumorigenesis and cell differentiation blockade (Telloni, 2017). In 1960, Pierce et al. demonstrated the self-differentiation of teratoma cells *in vivo* and *in vitro*, causing tumors to develop in a benign direction. This was the first time the idea of limiting tumor progression by inducing cell differentiation was advocated (Lotem and Sachs, 1988). In 1971, Friend et al. discovered that dimethyl sulfoxide could induce differentiation in mouse erythrocyte cells, establishing a precedent for further studies on tumor differentiation-inducing therapy (Joshi et al., 1985). Since then, a series of differentiation regulators, including retinoic acids, granulocyte-macrophage colony-stimulating factor, and interleukin-3, have been used in several tumor types such as osteosarcoma, acute myeloid leukemia (AML), pancreatic cancer, liver cancer, and NB (de Thé, 2018).

### 2.3 Relationship between differentiation disorders and tumorigenesis

With increasing advances in cancer pathophysiology, the understanding of areas of carcinogenesis and cancer development has improved. Reportedly, cancer is mediated by the action of several pathways that causes normal cells to become tumorigenic and proliferate, leading to tumor growth (Hanahan and Weinberg, 2000; Hanahan and Weinberg, 2011). The development, differentiation, and organization of cells into tissues to assume homeostatic function are accompanied by final differentiation during organogenesis. The result of cell differentiation is non-proliferation in most cases, which is in contrast to the continuous proliferation required for tumorigenesis. Growing evidence suggests that cells acquiring more cellular phenotypic plasticity and avoiding end-differentiation play important roles in tumor development (Yuan et al., 2019). Cell phenotypic plasticity is defined as the cell’s ability to reprogram and change its phenotypic characteristics, primarily dedifferentiation, trans-differentiation, and differentiation block, which can occur during multiple life processes such as embryonic development, tissue regeneration, wound healing, and tumor formation (Neftel et al., 2019). Tumorigenesis and its malignant development can result from a disruption the normal differentiation of progenitor cells to mature cells in many ways (Hanahan and Weinberg, 2011; Neftel et al., 2019).

Dysfunction of cell differentiation is strongly related to tumor development, and the level of differentiation correlates with the prognosis for tumor development. A well-known example is NB, which is a tumor derived from the sympathetic adrenal lineage, where fewer cases are caused by somatic gene mutations and most are caused by impaired development of neuro crest cells (Cheung and Dyer, 2013; Matthay et al., 2016). Moreover, NB cells can differentiate into mature neurons spontaneously or following drug exposure (Raman and Raman, 2018). Additionally, it has long been established that chromosomal translocations are the primary cause of acute promyelocytic leukemia (APL), these translocations prevent myeloid progenitor cells from undergoing terminal differentiation, owing to which the cells develop into granulocytes (Warrell et al., 1993; He et al., 1999).

An inverse association has been demonstrated between tumor cell differentiation and prognosis in several cancer types. As a result, therapies that induce tumor cell differentiation, to some extent, have been developed. For example, in NB, the type of differentiation affects the prognosis of patients (Maris, 2010). For patients with a well-differentiated tumor type and other biologic factors indicating a good prognosis, there exists a possibility of spontaneous regression of the cancer (Matthay, 1998). Even if cancer metastasizes to the liver, skin, and bone, low-dose radiotherapy, chemotherapy, or observational therapy can achieve a cure rate of >90% in this group of patients (Pinto et al., 2015). Conversely, patients with poorly differentiated types have poor outcomes, even after undergoing high-intensity treatment strategies, with a 5-year survival rate of <50% (Ladenstein et al., 2017; Amoroso et al., 2018).

## 3 Status of differentiation-inducing therapy

Compared with traditional cancer treatments such as chemotherapy, differentiation-inducing therapy is still in the early stages of treating malignant tumors. One reasons for this is a lack of recognized diagnostic targets or markers for inducing differentiation.

### 3.1 Application of retinoic acid in differentiation-inducing therapy

It is well-known that vitamin A promotes eye health. In addition, retinoic acid (RA; active form of vitamin A) exhibits antioxidant effects and gene transcriptional regulatory effects. It additionally maintains epithelial cell structure, cell growth, and cell differentiation (Takahashi et al., 2022). Liver, serving as storage organ of retinoids in body, regulates the retinol level in plasma (Chlapek et al., 2018). RBP4 transports retinol during cellular uptake and efflux, through receptors stimulated by retinoid acid 6 (STRA6). Retinol could be enzymatically activated to retinal, and which is then oxidized into retinoic acid by the isoforms of aldehyde dehydrogenase 1 (ALDH 1), specifically ALDH1A1, ALDH1A2, and ALDH1A3. The main transport way of retinol acid through the cell and into nucleus employs cellular retinoic acid binding proteins (CRABP) (Chlapek et al., 2018). When ligands existing, retinoic acid delivered by CRABP to retinoic acid receptor (RAR) or retinoic X receptor (RXR) or to others such as peroxisome proliferator-activated receptors (Chlapek et al., 2018). The expression of over 500 genes is up or downregulated by RA (Mezquita and Mezquita, 2019). Then retinoic acid regulates cellular activities including differentiation (Dobrotkova et al., 2018).

Members of the retinoid family, including vitamin A and its derivatives, such as 13-cis retinoic acid (13-*cis* RA) and all-trans-retinoic acid (ATRA), are well-established in the differentiation-inducing therapy. As we stated before, RA induces differentiation through RA receptors by recognizing and binding to DNA sequences to initiate target gene transcription (Takahashi et al., 2022). RA is tuned by the heterodimers formed by RAR and RXR, then targeting at many genes by RAR or RA itself. For instance, BTG2 is a direct target of RAR, induced by RA, which induces neuronal differentiation (Janesick et al., 2015). In addition to BTG2, RA potentially facilitates other genes, such as ERF, ETV3, which all participate in cell cycle and differentiation (Janesick et al., 2015).

It is discovered that the characteristic t(15:17) translocation of APL is within the locus encoding RARα, which results in the generation of the fusion of PML and RARα (Giguère and Evans, 2022). Several studies have assessed the role of ATRA as a differentiation-inducing therapy in APL (Huang et al., 1988; Warrell et al., 1991). Although ATRA therapy is currently considered the most effective treatment for APL, the exact molecular mechanism underlying its effect is unknown. APL is considered a promyelocytic leukemia protein (PML)-retinoic acid receptor-a (RARa) derived cancer, and the majority of APL cases are induced by a specific t(15,17) chromosomal translocation encoding the PML–RARα fusion protein (de Thé, 2018). This tumor protein is produced by the fusion of retinal, a gene on chromosome 17, and PML on chromosome 15 (Kakizuka et al., 1991). PML-RARa has been identified as one of the targets for APL to induce differentiation using ATRA (Kakizuka et al., 1991). RA targets APL by binding the RARa of the PML-RARa fusion protein, resulting in the dissociation of co-repressors and subsequent differentiation, thus driving complete remission (de Thé, 2018). In addition, APL research has identified other RARa fusion genes including, PLZF on chromosome 11, NPM on chromosome 5, and NuMA on chromosome 11 (Melnick and Licht, 1999). These findings, therefore, have important implications for identifying potential biomarkers of APL before using ATRA therapy and further promote the development of differentiation-inducing therapy in APL.

In addition to vitamin A derivative, retinal acid, a cocktail of ATRA, TGFβ inhibitors, GSK3β inhibitors, and H3K9 methyltransferase/G9a inhibitors have been successfully used to induce differentiation, including hepatoma cell lines, primary hepatoma cells, liver cancer stem cells, and drug resistant hepatoma cells (Zhang et al., 2022a). Similarly, studies have revealed that using RA in breast adenocarcinoma can promote breast cancer stem cell differentiation and loss of self-renewal ability as well as block the stemness of cancer cells, which reduces their invasion and metastasis (Biswas et al., 2019). In addition, Roghayeh et al. reported that culturing breast cancer cells using senescent cell culture supernatant and fibroblast supernatant can downregulate the expression of stem markers such as OCT4, SOX2, NANOG, and C-MYC, result in enhanced differentiation of breast cancer cells (Pourbagher et al., 2020).

Furthermore, vitamin A derivatives can suppress the proliferation of NB cells while promoting neurite formation, which is another example of differentiation induction therapy in solid tumors. Reportedly, pretreatment of NB cells with tretinoin before inoculation reduces tumor growth rate and volume as well as significantly reduces tumor formation rate, compared with control treatment (Abemayor et al., 1990; Ni et al., 2019). In patients with minimal residue high-risk NB, the use of 13-*cis* RA can improve the progression-free survival rate of patients by approximately 15%(Massó-Vallés et al., 2020). Reportedly, silencing MYCN promotes NB differentiation, and thus, maintenance therapy with 13-*cis* RA can downregulate MYCN expression and induce neuronal differentiation in NB cells(Massó-Vallés et al., 2020).

### 3.2 Role of MYCN in cell differentiation

MYCN gene, as previously stated, plays a key regulatory function in the genesis and progression of neuroblastoma. Members of the oncogene MYC family include c-MYC, N-MYC and L-MYC. c-MYC, called cellular MYC, was discovered as the cellular homologue of v-myc. L-MYC, called lung carcinoma derived homolog MYC, is located on chromosome 1 (1p32). MYCN (or N-MYC), called neuroblastoma derived homolog MYC, is located on chromosome 2 (2p24) (Massó-Vallés et al., 2020). MYC family members are transcription factors for the expression of many target genes, which in turn regulate the basic life course of cells, including functions such as cell proliferation, protein synthesis, metabolism, apoptosis, and differentiation (Kohl et al., 1983; Schwab et al., 1983; Eilers and Eisenman, 2008). The MYC proteins bind to E-box sequences (CACGTG) in a heterodimeric complex with Max. This dimer recruits a lot of transcriptional factors, then inducing differentiation (Westermark et al., 2011).

Amplification of the MYCN gene is one of the few indicators of poor prognosis in neuroblastoma, and MYCN amplification is common in individuals with high-risk NB, accounting for roughly 20% of primary NB (Schnepp and Maris, 2013). Even in NB patients with other favorable prognosis, MYCN gene amplification predicts poor mortality in 15%–35% of patients (Seeger et al., 1985; Westermann et al., 2008). Furthermore, patients with high-risk NB who do not have MYCN amplification frequently have higher c-MYC expression (Westermann et al., 2008). All of these indicates that MYCN signaling is crucial in maintaining an undifferentiated phenotype, and MYCN can be used as one of the basis and evaluation indicators for the application of 13-cis RA-induced differentiation treatment for neuroblastoma.

As we stated before, amplification of MYCN results in cancer malignancies such as cell cycle arrest in G1 phase and morphological differentiation, so targeting MYCN is a promising therapy (Westermark et al., 2011). The MYCN pathway could be targeted in different ways to go through differentiation-inducing. The use of RNA interference targeting MYCN is used as a clinical strategy, and it is found that some molecules interfering with the c-MYC-Max could induce differentiation (Westermark et al., 2011). In addition, targeting downstream of MYCN pathway is a reliable way to induce differentiation.

NDRG2, the downstream regulatory gene of MYCN, is a member of the NDRG family and plays a crucial role in controlling the differentiation and proliferation of colorectal cancer (CRC) cells (Derwinger et al., 2010). Reportedly, CRC is characterized by aberrant differentiation, and CRC with poor differentiation is more aggressive and has a higher proliferative and metastatic capacity, which contribute to a higher negative impact on survival and prognosis (Bienz and Clevers, 2000). NDRG2 is a tumor suppressor, that is, downregulated in a variety of malignancies and is associated with the tumor differentiation stage (Lorentzen et al., 2007). It has been demonstrated to induce tumor cycle arrest by blocking the activity of the E3 ligase Skp2, thereby driving the differentiation of CRC cells. This suggest that NDRG2 may be a critical biomarker for CRC differentiation (Shen et al., 2018).

Poorly differentiated HCC is characterized by liver progenitors, is typically aggressive, and has a poor clinical prognosis (Lee et al., 2006). In their study, Qian Yan et al. developed an *in vitro* hepatocyte differentiation model to simulate liver development and HCC progression (Liu et al., 2020). They found that high expression of PGC7/DPPA3, a member of the developmental pluripotency-associated protein family, can promote HCC cell dedifferentiation and maintain an epigenetic status suitable for liver progenitor cells, resulting in liver cancer metastasis and poor prognosis. Reportedly, PGC7 and GLI1/MYCN are positively correlated with differentiation markers. Therefore, inhibiting PGC7/GLI1/MYCN may reverse the poorly differentiated hepatoma cells, inducing a potential therapeutic strategy of differentiated-inducing therapy in patients with liver cancer (Yan et al., 2021).

MYCN modulation has additionally been documented in the literature in neuroendocrine prostate cancer (NEPC), rhabdomyosarcoma, and glioma differentiation therapy (Estiar et al., 2017; Liu et al., 2019; Laubscher et al., 2021). Roghayeh et al. discovered that the downregulation of c-MYC expression in breast cancer cell culture by inducing higher differentiation of cancer cells (Pourbagher et al., 2020). More than 70% of human cancers have abnormal MYC expression, which is associated with poor prognosis and aggressiveness (Pelengaris et al., 2002; Vita and Henriksson, 2006). Therefore, owing to the characteristics of MYC overexpression in malignancies and its widespread role in transcription regulation, it is regarded as an ideal therapeutic target. Because the MYC family performs a dual role in normal and malignant cells, targeting the MYC protein is difficult. Furthermore, because antibodies to MYC are not easily available due to the lack of typical small molecule-bound enzyme capsules and MYC location in the nucleus, no specific medicines can directly target MYC (Wang et al., 2021).

### 3.3 Other targets and markers of cell differentiation

Although MYC protein is involved in several physiological processes, such as cell differentiation, it cannot be used to promote differentiation in most tumors. Thus, there is a lack of gold standards for differentiation markers, and this topic has piqued the curiosity of researchers.

Histone deacetylase (HDAC) is a large class of epigenetic metalloenzyme that are involved in gene transcription and regulation; proliferation, differentiation, migration, death and angiogenesis (Guerriero et al., 2017). Following extensive research on their role in anti-tumor proliferation, HDAC inhibitors are being utilized in tumor treatment. Specifically, HDAC1 and HDAC2 have been reported to play roles in NB cell differentiation. A few HDAC inhibitors do not have a high differentiation-inducing effect when administered alone; therefore, they are combined with 13-*cis* RA to considerably increase their differentiation activity (Coffey et al., 2001; Rettig et al., 2015). Furthermore, selective differentiation therapy with some HDAC inhibitors has been documented for breast cancer, liver cancer, and AML (Ji et al., 2019; Mody et al., 2021; Salmon et al., 2022).

Bromodomain-containing 4 (Brd4) belongs to the bromine-containing domain and the Bromodomain and Extraterminal family and regulates cell transcription by interacting with acetylated histones (Filippakopoulos et al., 2010). Brd4 acts as an oncogene in the development of cancers, such as squamous cell carcinoma, leukemia, CRC, and breast adenocarcinoma by detecting DNA damage, activating, repairing, and maintaining telomeres (French et al., 2003; Wu and Chiang, 2007). Reportedly, in AML, inhibition of Brd4 causes end-myeloid differentiation and leukemia stem cell elimination (Zuber et al., 2011). Moreover, the Brd4 inhibitor ZBC-260 has been found to downregulate the expression of c-MYC, Bcl2, and c-MYC-related genes to reduce pituitary tumorigenesis (Shi et al., 2020). Shan Zeng et al. demonstrated that Brd4 plays an important role in plasma cell differentiation by regulating the expression of B lymphocyte-induced maturin 1 (Liu et al., 2018).

The enhancer of zeste homolog 2 (EZH2) is a catalytic component of multicomb group protein 2, that is, involved in hematopoietic stem cell proliferation and differentiation, thymogenesis, and lymphomatogenesis (Yamagishi and Uchimaru, 2017). EZH2 mutations are associated with the development of gastric, breast, prostate, and liver cancers as well as melanoma. Moreover, EZH2 suppression or knock-out causes significant alterations in neurite extension and upregulates neuronal differentiation markers, which enhance the expression of tropomyosin receptor kinase, and further induce NB cell differentiation (Li et al., 2018). Berberine inhibits cell proliferation and accelerates NB cell differentiation by inhibiting EZH2 expression (Naveen et al., 2016). Moreover, EZH2 suppresses proliferation checkpoint genes and generates a bivalent chromatin domain at the critical regulatory sites to temporarily limit germinal center B cell development (Béguelin et al., 2013).

The tyrosine kinase receptor regulates several of biological activities such as proliferation, differentiation, migration, and metabolism (Lemmon and Schlessinger, 2010). The overexpression or activation mutations of ALK frequently cause malignant proliferation and differentiation disorders in tumor cell (Carén et al., 2008; Heukamp et al., 2012). Reportedly, ALK mutations or amplifications have been found in approximately 14% of high-risk patients with NB, and a few studies have shown that ALK alone or in combination with other agents can induce NB differentiation (Brodeur et al., 2009). In addition, considering ALK stimulates central and peripheral nerve development, ALK inhibitors have been found to play a role in the maturation of non-small cell lung cancer because (Defaye et al., 2022). As plasma cell differentiation differs between diffuse large B-cell lymphoma and multiple myeloma, as well as a greater chance of MYC and ALK mutations, the inhibitors to stimulate plasma cell differentiation are available (Montes-Moreno et al., 2012). NEPC has a poor prognosis, is an aggressive subtype of prostate cancer, and is often accompanied by MYCN application. In contrast, both ALK activation and MYCN amplification are well-known drivers in NB. However, the mechanism and therapeutic targets of NEPC are comparable to those of NB. Unno et al. (2021) discovered that the co-activation of ALK and MYCN induces NEPC by inducing the Wnt/β-catenin pathway, which is a major target for triggering differentiation.

Given the enormous number of cancers associated with differentiation, it is surprising that MYCN and other contemporary differentiation-related markers are not being widely used. It is additionally surprising, as these markers overlap in various malignancies. As a result, it is critical to understand the process of tumor cell differentiation and to seek both the gold standard for cell differentiation as well as serological or histologic indications of malignancy.

## 4 Mechanism of differentiation of malignant tumors

### 4.1 Cell cycle and differentiation

Several studies have revealed that the cell cycle regulates stem cell differentiation (Engström, 2021). Cell terminal differentiation is intimately linked with the cell cycle, particularly with transition in dividing cells (Soufi and Dalton, 2016). The cell cycle is divided into four stages: gap 1 (G1) stage, DNA synthesis (S) stage, gap 2 (G2) stage, and mitosis (M) stage. The passage between mitosis and the G1 phase allows for differentiation (Engström, 2021). After receiving signals, stem cells undergo alterations in the G1 phase (Dalton, 2015; Gao and Liu, 2019), losing pluripotency throughout the S and G2 phases (Gao and Liu, 2019). The progression of the G1 phase is considered one of the mechanisms of differentiation regulation (Ruijtenberg and van den Heuvel, 2016). A prolonged G1 phase is thought to promote the components required for cell differentiation, whereas a short G1 phase limits the influence of differentiation signals and leads to pluripotency (Dalton, 2015; Liu et al., 2019). Compared with somatic cells, stem cells have a distinct cell cycle characterized by a quick cycle and a brief G1 phase (Kareta et al., 2015). In mammals, undifferentiated cells have an unusual cell cycle with a short G1 phase; they even lack G1 and G2 phases that are seen in some other animals, such as flies, frogs and zebrafish (Ruijtenberg and van den Heuvel, 2016).

Cyclins and a variety of cyclin-dependent kinases (CDKs), which phosphorylate the substations during the cell cycle, regulate all stages of cell cycle progression (Soufi and Dalton, 2016; Wood and Endicott, 2018; Liu et al., 2019). The knockdown of CDK1, CDK2, cyclin E, or B1, as well as the treatment of CDKI lead to cell differentiation and loss of pluripotency (Liu et al., 2019). During the G1 phase, CDKs govern cell cycle progression by phosphorylating RB family proteins during the restriction (R-) point (Soufi and Dalton, 2016). Mitogens and growth factors can induce the expression of D-type cyclins (cyclin D) and activate CDK4 or CDK6, thus regulating the cell cycle (Ruijtenberg and van den Heuvel, 2016). Cyclins bind, activate, and provide substrate specificity to CDKs and then form the CDK-cyclin complex (Liu et al., 2019). CDK4/6-cyclin D hypophosphorylates the members of the RB tumor suppressor protein family, thus reducing the repression level of E2F/DP, which is correlated with pRb (Ruijtenberg and van den Heuvel, 2016). The commencement of E2F transcription induces cyclin E and additional cyclin genes (Ruijtenberg and van den Heuvel, 2016). Subsequently, cyclin E binds to CDK2 and further phosphorylates pRb, releasing E2F and causing the cells to enter the S phase (Ruijtenberg and van den Heuvel, 2016). Except for cyclins and CDKs, all other biomarkers regulate differentiation by acting on the cell cycle (Ruijtenberg and van den Heuvel, 2016). Specifically, the CDK inhibition protein (CDKI) can prevent cells from progressing from the G1 to S phase (Ruijtenberg and van den Heuvel, 2016). In most cases, CDKI overexpression suppresses CDK during the G1 phase, as well as the hypophosphorylation of RB family proteins, which decreases E2F target genes and leads to terminal differentiation (Soufi and Dalton, 2016). For example, PINK inhibitors, like p15 and p16, are a type of CDKI that bind specifically to CDK4/6 to block its activation (Ruijtenberg and van den Heuvel, 2016). Another type of CDKI, the Cip/Kip family (includes p25 and p27) has a negative effect on the regulation of CDK2-cyclin E (Ruijtenberg and van den Heuvel, 2016). The balance of CDK and transcription factors that influence the cell cycle dictates how cells are fated (Soufi and Dalton, 2016). Upstream E3 ligases, including the Anaphase Promoting Complex/Cyclosome in collaboration with the FZR1/Cdh1 coactivator, and Skp1, Cullin, F-box factor (SCF) complexes, control the cell cycle by ubiquitin-dependent protein degradation (Ruijtenberg and van den Heuvel, 2016). When combined with FBW7, cyclin E degenerates and inhibits cell cycle progression (Ruijtenberg and van den Heuvel, 2016). However, when SCF binds to Skp2, it degrades p21 and p27 and promotes entry into the cell cycle (Ruijtenberg and van den Heuvel, 2016).

CDKs repress the differentiation transcription factors MYOD and NGN2 during skeletal myogenesis and neurogenesis, allowing cells to proliferate while limiting their differentiation. Additionally, blocking CDKs was found to induce the spontaneous differentiation of pluripotent stem cells (PSCs) (Soufi and Dalton, 2016). Cyclins were previously thought to assist CDK in cell differentiation; however, they can govern cell differentiation independently (Wood and Endicott, 2018). Treatment with CDK1, CDK2, cyclin E or B1, and CDKI all result in differentiation (Liu et al., 2019). Furthermore, it has been found that cell cycle regulation is not limited to the G1 phase; the G2 phase additionally affects cell cycle and differentiation regulation.

Evidence suggests that in AML, the cell cycle is correlated with cancer cell differentiation; the early stage of cell development is inhibited, which results in cancer development (Hu and Zuckerman, 2014). Reportedly, ATRA and vitamin D3 induce AML differentiation, that is, accompanied by the downregulation of CDK2, CDK6, c-MYC, and cyclin E and the upregulation of a series of CDKIs as well as a high level of pRb, especially p21 and p27 (Hu and Zuckerman, 2014). The downregulation of c-M can inhibit the G0/G1 phase, downregulate CDKs and upregulate p27, all of which inhibit cell differentiation (Hu and Zuckerman, 2014). Moreover, pRb can promote the differentiation of leukemia cells (Hu and Zuckerman, 2014). Furthermore, CDK-activating kinase (CAK) can interact with RARa, and the inhibition of CAK by ATRA leads to hypophosphorylation of AML-RARa, which finally results in APL cell differentiation (Hu and Zuckerman, 2014).

### 4.2 Hypoxia and cell differentiation

In addition to affecting the cell cycle, hypoxia has been shown to influence cell differentiation. Hypoxia is a clinical condition that can lead to cellular dysfunction and death as well as activate molecular pathways in multiple stem cells, promoting their state of differentiation (Mitroshina et al., 2021). Reportedly, pluripotent stem cells and differentiated cells differ with regard to a lack of traits such as low ATP/cell content and a high rate of oxygen consumption (Atashi et al., 2015; Bao and Wong, 2021). Tissue hypoxia plays a crucial role in the development of stem cells; it facilitates embryonic stem cells (ESC) and induced pluripotent stem cells (iPSC) (Podkalicka et al., 2020). It has been demonstrated that hypoxia inhibits the differentiation of hESCs (Vieira et al., 2011), as well as maintains an undifferentiated state of cancer cells, and promotes the formation and maintaining of CSCs (Yang et al., 2020). In NB and breast tumor cells, hypoxia induces de-differentiation to a stem cell-like phenotype (Mohlin et al., 2017). Thus, hypoxia plays an important role in maintaining the differentiation state of stem cells and blocking their differentiation.

Hypoxia influences the differentiation primarily through two mechanisms: hypoxia inducible factor 1 (HIF) family modulation and reactive oxygen species (ROS) production. HIF, a vital regulator of hypoxia, is a heterodimer composed of α and β subunits that regulate over 700 genes, with HIF-1α primarily modulating differentiation in hypoxic conditions (Podkalicka et al., 2020; Mitroshina et al., 2021). Hypoxia can additionally induce HIF-1α, which is essential for reacting to low oxygen stress via a variety of transcriptional programs (Palazon et al., 2014; Xie et al., 2019), including proliferation, migration, and differentiation (Hadanny and Efrati, 2020). In contrast, HIF levels normalize following the regulation of oxygen conditions by a few molecules. In low-oxygen settings, the prolyl hydroxylase domain (PHD) protein is stable and functions as a transcription factor that control genes in oxygen-depleted conditions and mediates O2-dependent degeneration of HIF-1a (Lin et al., 2006). However, HIF-1a can be hydroxylated and degraded by PHD under normal oxygen levels (Hadanny and Efrati, 2020). Factor inhibiting HIF is another factor that inhibits the transcriptional activity of HIF-1α by hydroxylation (Cejudo-Martin and Johnson, 2005). Generally, hypoxia activates HIF-1α by inducing differentiation regulation. Under hypoxic settings, HIF-α stabilizes and heterodimerizes with HIF-β, which activates HIF transcription factors. These factors induce gene expression after binding to the hypoxia response element, which includes the vascular endothelial growth factor receptor (VEGFR) and its upregulated receptors (Podkalicka et al., 2020). This result in early osteogenic differentiation and vascular differentiation of ESCs/iPSCs (Podkalicka et al., 2020; Zhang et al., 2022b). Additionally, the negative regulator c-MYC is induced under severe and mild hypoxia and is regulated by HIF-1a (Podkalicka et al., 2020). The knockdown of HIF-2a has been found to downregulate CXCR4, OCT4, SOX2, and NANOG, which are vital transcription factors for pluripotency, and function along with the upregulation of the differentiation marker SSEA1 (Lin et al., 2006; Podkalicka et al., 2020). Thus, in hypoxic conditions, deficiency of HIF suppressors induces HIF-1a hydroxylation, resulting in the overexpression of transcription factors, such as c-MYC and VEGR, to regulate differentiation.

ROS is an oxygen-derived small molecular involved in progression of cancer cells, mainly from mitochondrial electron transport systems, such as NADPH oxidases, xanthine oxidase, cytochrome P450, nitric oxide synthases, lipoxygenases, heme oxygenase, cyclooxygenases, myeloperoxidase, and monoamine oxidases (Atashi et al., 2015). In solid tumors, hypoxic tissue promotes the formation of ROS, with evidence indicating that ROS has an effect on cell differentiation (Ebrahimi et al., 2020). *In vitro*, ROS is considered as a second messenger to regulate downstream signal cascades, including PI3K/AKT pathways, for inhibiting differentiation and cell cycle (Feng et al., 2019). Reportedly, ROS production influences MSC differentiation; suppresses Wnt/b-catenin, MAPK (NELL-1), and Hh signaling that control osteoblast differentiation; and induces adipogenesis by FOXO, PPARγ, and CEBPs signaling (Atashi et al., 2015).

### 4.3 Metabolism and cell differentiation

Metabolic pathways are critical areas of cell differentiation and provide signals to the stem cells for self-renewal and differentiation (Hu et al., 2016; Intlekofer and Finley, 2019). Reportedly, mitochondrial morphology and the transition from glycolysis to mitochondrial oxidative phosphorylation (OXPHOS) are characteristics of the state of differentiation (Hu et al., 2016; Sancho et al., 2016; Intlekofer and Finley, 2019). In addition, differentiation is regulated by glycolysis, OXPHOS and metabolites that regulate epigenetic changes (Shyh-Chang and Ng, 2017).

Cells that rapidly undergo proliferation, such as cancer and stem cells, undergo aerobic glycolysis to meet their energy requirement. This process, termed, the “Warburg effect”, provides cancer cells their pluripotency (Intlekofer and Finley, 2019). During differentiation, the amount of glucose, that is, catabolized by OXPHOS increases in proportion to that produced by the mitochondria. Conversely, metabolism additionally reverts from glucose oxidation by OXPHOS to major glycolysis during the reprogramming of somatic iPSCs (Kilberg et al., 2016). Glycolysis can be interrupted to alter the status of cancer cell differentiation. Specifically, reduced glycolytic lactate generation in the early stage reprograms differentiated cells to pluripotent cells (Intlekofer and Finley, 2019). Furthermore, lower glycolytic flow and decreased production of glycose-derived acetyl-CoA results in a rapid lowering of histone acetylation, resulting in a pluripotent state (Intlekofer and Finley, 2019).

As previously stated, mitochondrial ROS play a role in cell differentiation. As a metabolite, low levels of mitochondrial H_2_O_2_, an ROS, contributes to maintaining the pluripotency of PSCs, whereas high levels of H_2_O_2_ induce cell differentiation (Chakrabarty and Chandel, 2021). Like H_2_O_2_, ROS has a similar effect on different stem cells, including spermatogonial stem cells, neural stem cells (NSCs) and airway basal stem cells (Chakrabarty and Chandel, 2021). Certain metabolic changes improve the methylation status of DNA (Intlekofer and Finley, 2019). Cancer-related DNA hypermethylation and repressive histones suppress the gene involved in cell differentiation (Intlekofer and Finley, 2019; Chakrabarty and Chandel, 2021). For example, both S-adenosylmethionine (SAM) and branched chain amino acid transaminase (BCAT1) cause differentiation suppression. SAM directly influences methylation, and, BCAT1 overexpression depletes a-KG, which limits ten-eleven-translocation (TET) function that results in DNA methylation (Intlekofer and Finley, 2019). Moreover, lower threonine levels and the inhibition of threonine dehydrogenase might reduce SAM pools and methylation, resulting in altered differentiation (Intlekofer and Finley, 2019). In addition, several other metabolism-related mechanisms affect cell differentiation, and these include mitochondrial tricarboxylic cycle metabolism and electron transport chain (ETC) function; however, the exact mechanism underlying their effect is unclear (Chakrabarty and Chandel, 2021). Proline metabolism affects ESC differentiation, and its downstream metabolites act as signaling molecules that participate in stem cell differentiation (Kilberg et al., 2016). However, specific mechanisms require further exploration (Chakrabarty and Chandel, 2021).

## 5 Status of research on differentiation-inducing therapy

In 1960, Pierce et al. identified the self-differentiation ability of teratoma cells, paving the way for the investigation of differentiation-inducing treatment (Jin et al., 2020).Since then, research on specialized therapeutic medicines such as RA, cAMP, sodium butyrate and cytokines has made significant progress (de Thé, 2018). The development of differentiation inducers has made significant progress because of research into differentiation induction treatment. Differentiation-inducing therapy has become the standard treatment for APL, with cure rates exceeding 90% (Jin et al., 2020). Moreover, Differentiation therapy has proven beneficial in the treatment of NB and other malignancies (Jin et al., 2020). The medicines and targets researched for differentiation-inducing treatment are further discussed below.

One of the earliest differentiation inducers used was RA, an oxidative carotenoid generated from vitamin A (Dobrotkova et al., 2018). RAR and RXR are the two receptor families that bind to co-hinders to suppress downstream signaling in the absence of ligands (Jin et al., 2020). Binding of RA to RAR-RXR heterodimer induces differentiation (Jin et al., 2020). In addition, the derivatives of RA, such as ATRA, 9-*cis* RA, and 13-*cis* RA, have been assessed for their role as differentiation inducers (Takahashi et al., 2022). Differentiation-inducing treatment with RA has been examined in clinical trials and observational studies for several tumors, including APL, breast cancer, B-cell lymphoma, cervical carcinoma, Ewing’s sarcoma, cutaneous T-cell lymphoma, glioblastoma multiforme, hepatocellular carcinoma, glioma, and lung cancer (Lippman and Meyskens, 1987; Villablanca et al., 1995; Muto et al., 1996; Toma et al., 2000; Duvic et al., 2001; Vaishampayan et al., 2005; Villablanca et al., 2006; Hu et al., 2009; Schneider et al., 2009; Arrieta et al., 2010; Moore et al., 2010; Zapletalova et al., 2012; Tassara et al., 2014; Nijhof et al., 2015; Penas-Prado et al., 2015; Basu et al., 2016; Cowan et al., 2017). Arsenic trioxide is another single medicine that has been used as a differentiation inducer, and functions by stimulating malignant cell differentiation via PML-RARa degradation (Jin et al., 2020). As we stated before, a large number of clinical trials of differentiation-inducing therapy emerged, which presented different results during different clinical trials. We have listed the recent clinical trials of common types of cancers here in Table 1.

**TABLE 1:** Overview of the recent clinical trials of common types of cancers.

Type of cancer	Drug action	Drug regimen	Type of treatment	Reference
AML	positive effect	ATRA + decitabine	phase II	Lübbert et al. (2020)
APL	positive effect	ATRA + ATO	phase III; multicenter	Platzbecker et al. (2017)
Breast carcinoma	positive effect	ATRA + paclitaxel	phase II	Bryan et al. (2011)
Cervical carcinoma	negative result	RA + IFN	phase II	Basu et al. (2016)
Hepatocellular carcinoma	prevention of relapse	polyprenoic (oral)	Observational study	Muto et al. (1996)
Hepatocellular carcinoma	negative result	ATRA (oral)	phase II	Meyskens et al. (1998)
Neuroblastoma	positive effect	13-cis RA	randomized study	Matthay et al. (1999)
Neuroblastoma	positive effect	13-cis RA	Randomized Controlled Trial	Matthay et al. (2009)
Non-small lung cancer	positive effect	ATRA + chemotherapy	phase II	Arrieta et al. (2010)
Ovarian carcinoma	positive effect	13-cis RA	phase II	Recchia et al. (2010)
Pancreatic carcinoma	positive effect	ATRA	phase I	Kocher et al. (2020)
thyroid cancer	positive effect	13-cis RA	Observational study	Simon et al. (2002)
Prostate carcinoma	negative result	ATRA	phase II	Culine et al. (1999)
Renal carcinoma	positive effect	13-cis RA + IFN	phase III	Aass et al. (2005)

Furthermore, agents that block dedifferentiation targets have been found to exert a positive effect on differentiation. MYCN inhibitors, such as aurora A kinases MLN8054 and MLN8237, have been used as differentiation treatments, with MLN8237 achieving total tumor remission in mouse models (Jin et al., 2020). The ALK inhibitors TAE684, Trk inhibitors, lestaurtinib, and the MEK inhibitor cobimetinib all have been found to promote fractionation (Jin et al., 2020).

However, there are some disagreements regarding differentiation-inducing treatments. The majority of patients who receive differentiation-inducing treatment acquire resistance (Chlapek et al., 2018). Furthermore, differentiation-inducing agents, such as targeted medicines, have limitations (Jin et al., 2020) They only benefit a tiny percentage of patients, with “off-target” effects restricting their usage (Jin et al., 2020). In addition, ATRA and 13-*cis* RA are pan-RAR activators that draw attention away from the side effects of differentiation inducers. The long-term administration of natural retinoids is restricted by vitamin A toxicity, including liver and lipid abnormalities, dry skin, teratogenicity, and bone and connective tissue damage (Dobrotkova et al., 2018). Moreover, rather than reducing tumor development, differentiation inducers activate peroxisome proliferator-activated receptors, boost tumor mitosis, and induce anti-apoptotic activity, and indirectly promote tumor proliferation (Takahashi et al., 2022). In some cell types, RA antagonize cell differentiation and promote stemness, instead of induce cell differentiation (Mezquita and Mezquita, 2019). As a result, further investigation and development are required for differentiation inducers.

## 6 Summary and outlook

In conclusion, differentiation-inducing therapy may not only be a successful treatment but may also aid in identifying the root cause of tumor heterogeneity. Initial research has identified several targets and indicators linked with differentiation and has demonstrated the potential of differentiation in cancer treatment, which offers a foundation for the development of differentiation-inducing therapy. However, there exists no obvious and effective therapeutic target. Further investigation into the transfer of cell epigenetics is required to better understand the mechanism underlying cell differentiation. Research in the field of differentiation will focus on how to overcome the heterogeneity of various tumor cells, identify common or universal differentiation markers, induce the differentiation of various tumor cells, and further develop differentiation inducers to ensure the safety of differentiation-inducing therapy.
